# Opportunities and challenges of self-binding directives: A comparison of empirical research with stakeholders in three European countries

**DOI:** 10.1192/j.eurpsy.2023.2421

**Published:** 2023-06-09

**Authors:** Matthé Scholten, Simone A. Efkemann, Mirjam Faissner, Marleen Finke, Jakov Gather, Tania Gergel, Astrid Gieselmann, Lia van der Ham, Georg Juckel, Laura van Melle, Gareth Owen, Sarah Potthoff, Lucy A. Stephenson, George Szmukler, Astrid Vellinga, Jochen Vollmann, Yolande Voskes, Anna Werning, Guy Widdershoven

**Affiliations:** 1 Institute for Medical Ethics and History of Medicine, Ruhr University Bochum, Bochum, Germany; 2 Department of Psychiatry, Psychotherapy and Preventive Medicine, LWL University Hospital, Ruhr University Bochum, Bochum, Germany; 3 Institute of Psychiatry, Psychology and Neuroscience, King’s College London, London, UK; 4 Department of Psychiatry and Psychotherapy, Charité Campus Benjamin Franklin, Berlin, Germany; 5 Department of Ethics, Law and Humanities, Amsterdam UMC, Vrije Universiteit Amsterdam, Amsterdam, The Netherlands; 6 GGZ inGeest, Amsterdam, The Netherlands; 7 Mentrum/Arkin, Amsterdam, The Netherlands

**Keywords:** Advance statement, crisis plan, psychiatric advance directive, self-binding directive, Ulysses arrangement

## Abstract

**Background:**

Self-binding directives (SBDs) are psychiatric advance directives that include a clause in which mental health service users consent in advance to involuntary hospital admission and treatment under specified conditions. Medical ethicists and legal scholars identified various potential benefits of SBDs but have also raised ethical concerns. Until recently, little was known about the views of stakeholders on the opportunities and challenges of SBDs.

**Aims:**

This article aims to foster an international exchange on SBDs by comparing recent empirical findings on stakeholders’ views on the opportunities and challenges of SBDs from Germany, the Netherlands, and the United Kingdom.

**Method:**

Comparisons between the empirical findings were drawn using a structured expert consensus process.

**Results:**

Findings converged on many points. Perceived opportunities of SBDs include promotion of autonomy, avoidance of personally defined harms, early intervention, reduction of admission duration, improvement of the therapeutic relationship, involvement of persons of trust, avoidance of involuntary hospital admission, addressing trauma, destigmatization of involuntary treatment, increase of professionals’ confidence, and relief for proxy decision-makers. Perceived challenges include lack of awareness and knowledge, lack of support, undue influence, inaccessibility during crisis, lack of cross-agency coordination, problems of interpretation, difficulties in capacity assessment, restricted therapeutic flexibility, scarce resources, disappointment due to noncompliance, and outdated content. Stakeholders tended to focus on practical challenges and did not often raise fundamental ethical concerns.

**Conclusions:**

Stakeholders tend to see the implementation of SBDs as ethically desirable, provided that the associated challenges are addressed.

## Introduction

Psychiatric advance directives (PADs) are documents by means of which mental health service users can express their treatment preferences for future mental health crises [1, 2]. PADs receive strong support from service users [3, 4] and have been shown to significantly reduce involuntary hospital admissions [5, 6]. Despite having several concerns [7, 8], most clinicians endorse PADs [9, 10], especially when they are involved in the drafting process [11]. Clinicians’ concerns are mitigated, moreover, by the fact that the content of PADs is generally clear and compatible with professional standards [12].

Self-binding directives (SBDs) are PADs that include a clause in which service users consent in advance to involuntary hospital admission and treatment under specified conditions [13, 14]. They are also often referred to as Ulysses contracts or arrangements, referring to Homer’s Ulysses, who was able to resist the lure of the Sirens on his journey home to Ithaca by instructing his crew to tie him to the mast of the ship and to ignore his entreaties to be released.

SBDs are useful in mental disorders that involve fluctuating mental capacity and anticipated treatment refusals during mental health crises, notable examples of which are psychotic and bipolar disorders [13]. Notwithstanding small variations in criteria across jurisdictions, mental capacity refers to the functional ability to make treatment decisions based on one’s own fundamental values and preferences [15, 16]. During mental health crises involving impaired mental capacity, persons sometimes make decisions that they would not have made had they had mental capacity. Such decisions regularly involve refusal of hospital admission and treatment and can have far-reaching consequences. Since SBDs enable service users to instruct clinicians to overrule such refusals, they are a vital part of advance care planning in mental health care.

Potential opportunities and challenges of SBDs have been discussed in the ethics and legal literature. Discussed opportunities include the promotion of service user autonomy, facilitation of early intervention, prevention of harm, promotion of well-being, and improvement of the therapeutic relationship [13, 14, 17–25]. Discussed challenges include the possibility of undue influence during SBD completion, increase of coercion due to premature SBD activation, invalidity of SBDs due to a lack of identity between past and present self, and invalidity of SBDs due to outdated consent [26–28]. Until recently, little was known about stakeholders’ views on the opportunities and challenges of SBDs [29–31].

This article aims to foster an international exchange on SBDs by comparing recent empirical findings on stakeholders’ views on the opportunities and challenges of SBDs from Germany, the Netherlands, and the United Kingdom.

## Methods

The current comparison is based on our interpretation of the findings from empirical stakeholder studies carried out between 2017 and 2021 by research teams at Ruhr University Bochum (Germany), VU University Amsterdam (the Netherlands), and King’s College London (the United Kingdom). Table 1 summarizes the characteristics of these studies.

**Table 1.** Characteristics of empirical studies

References	Sample	Data collection	Data analysis
Hindley et al. [32]	932 persons with bipolar	Online survey	Quantitative
Stephenson et al. [33]	10 persons with bipolar, 3 relatives, 19 professionals; 5 service user-led organizations, 5 mental health clinical teams	Focus groups; consultation process	Qualitative
Gergel et al. [34]	565 persons with bipolar	Online survey	Qualitative
Potthoff et al. [35]	6 persons with bipolar, 6 relatives, 5 professionals, 5 researchers	Focus group; semi-structured interviews	Qualitative
Stephenson et al. [36]	17 persons with bipolar, 14 relatives, 18 professionals	Semi-structured interviews	Qualitative
Van Melle et al. [37]	7 service users, 14 professionals	Semi-structured interviews	Qualitative
Werning et al. [38]	225 persons with bipolar, 105 relatives, 45 professionals	Online survey	Quantitative

The method of the current article is an empirically informed conceptual and ethical analysis, where comparisons were drawn using an interactive and collaborative expert consensus process. Experts had backgrounds in bioethics, medicine, nursing, philosophy, psychiatry, psychology, and the social sciences, and the group included both lived experience and clinical expertise.

Regular exchanges of research results between the research groups took place from 2019 onward. In June 2021, M.S. organized a workshop and brainwriting session with members of all teams using the online visual collaboration platform Miro to compare empirical data on stakeholders’ views. M.S. collected and structured the results of the session and included them in an online interactive document. Members of all teams added information to and provided feedback on this document in an online iterative feedback process from July to September 2021. The research teams discussed their empirical results at the SALUS Midterm Symposium on SBDs in September 2021. M.S. subsequently wrote the first version of the manuscript based on the information in the interactive online document and the input from the symposium. This manuscript was opened for feedback and additions as an online interactive document from October to December 2021. M.S. incorporated the feedback, distributed the penultimate draft among all coauthors in August 2022, and made final changes based on their feedback in December 2022.

## Results

In what follows, we describe important legal background differences between the three jurisdictions and summarize the key points of convergence that emerged from the findings on stakeholders’ perceptions of the opportunities and challenges of SBDs and the expert consensus process.

### Legal frameworks for SBDs

The differences between the legal frameworks for SBDs in the three jurisdictions are summarized in Table 2.

**Table 2.** The legal status of SBDs within the three jurisdictions

Jurisdiction	PADs legally binding	SBDs legally binding	Relevant legislation	Relevant sections
Germany	Yes	No	Guardianship law (part of the Civil Code; BGB); Mental health laws of the 16 states (PsychKHG)	Sec. 1827 and 1832 BGB
The Netherlands	Yes	Yes	Law on Compulsory Mental Healthcare (Wvggz)	Wvggz Sec. 4
England and Wales	No, if detained under MHA	No	Mental Capacity Act 2005 (MCA); Mental Health Act 1983 (MHA)	MCA Sec. 24

Abbreviations: PADs, psychiatric advance directives; SBDs, self-binding directives.

*The Netherlands:* Of the three jurisdictions, only the Netherlands has explicit legal provisions for SBDs. These are described in Article 4 of the Dutch Law on Compulsory Mental Health Care (*Wet verplichte geestelijke gezondheidszorg*; Wvggz). Service users who have mental capacity and are at least 16 years old can write an SBD in consultation with the treating mental health professional. Mental capacity must be assessed by an independent physician. SBDs must describe the circumstances in which involuntary treatment must be provided and give specific treatment instructions. They must also specify the maximum duration of involuntary treatment and the conditions under which it must be discontinued. A practical problem within the Dutch legal framework is that involuntary treatment based on an SBD is subject to a complex procedure for legal authorization which can take over 4 weeks time [14, 17].

*Germany:* While there are no specific legal provisions for SBDs in Germany, advance directives are legally binding and regulated in Section 1827 of the guardianship law in the German Civil Code (*Bürgerliches Gesetzbuch*; BGB). Advance directives apply to somatic and mental health conditions alike and can include both advance refusals and advance consent to medical interventions [39]. Although this goes some way in the direction of self-binding, any form of treatment against the current preferences of a person is subject to the criteria for involuntary treatment according to Section 1832 BGB [39]. Instructing professionals to provide involuntary treatment under self-prescribed conditions is thus not possible under German law.

*England and Wales:* While there are no legal provisions for SBDs in England and Wales, a model has been proposed which may support service users to make best use of the existing legislation [13, 33]. This model relies on the interface between the Mental Capacity Act 2005 (MCA) and the Mental Health Act 1983 (MHA). Service users can create an advance directive under the MCA which requests that an MHA assessment and involuntary hospital admission takes place when they have started displaying particular symptoms and their mental capacity is likely to be impaired. Two doctors (usually psychiatrists) and a specialist social worker are required to agree that hospital admission is necessary before it can be arranged, and they could use the advance directive to inform this assessment. The advance would accordingly have weight under the MHA Code of Practice.

### Opportunities of SBDs

Stakeholders perceived various opportunities of SBDs. Opportunities on which the studies converged are summarized in Table 3.

**Table 3.** Perceived opportunities of SBDs

Opportunities of SBDs
Promotion of autonomy
Avoidance of personally defined harms
Enabling early intervention
Reduction of admission duration
Improvement of the therapeutic relationship
Involvement of persons of trust
Avoidance of involuntary hospital admission
Addressing trauma
Destigmatization of involuntary treatment
Increase in professionals’ confidence
Relief for proxy decision-makers

Abbreviation: SBDs, self-binding directives.

*Promotion of autonomy:* SBDs can give service users more control over their life and treatment by enabling them to express and give force to treatment preferences and define the circumstances in which this treatment should be provided. Furthermore, drafting an SBD is a reflective process that can enhance service users’ self-understanding and self-management, for example, by creating a richer and shared understanding of personal relapse indicators and methods for crisis management. SBDs can also forge a more general sense of empowerment by ensuring that the voice of service users is heard and that service users are treated with dignity and respect.

*Avoidance of personally defined harms:* By triggering hospital admission and mental health treatment, SBDs can help service users to avoid harms that are important to them and defined by themselves. These harms can include health damage, financial damage, damage to personal projects, damage to personal relationships, and feelings of shame and guilt.

*Enabling early intervention:* Service users typically do not yet satisfy the criteria for involuntary hospital admission and treatment when they exhibit early warning signs. By allowing service users to personalize the criteria for involuntary hospital admission and treatment, SBDs can enable early intervention in mental health crises.

*Reduction of admission duration:* By enabling early intervention, SBDs can ensure that service users are admitted to hospital and treatment is initiated before their symptoms exacerbate. This can contribute to quicker recovery and hence to a reduction of the duration of admission.

*Improvement of the therapeutic relationship:* Drafting an SBD is a collaborative process in which service users and professionals share thoughts about the treatment preferences of service users, the medical aspects of their condition, and the expected benefits and risks of the available treatment options. They then jointly agree on and commit to a plan of treatment. This form of shared decision-making and mutual commitment can shape a relation of trust and improve the therapeutic relationship.

*Involvement of persons of trust:* SBDs imply the involvement of a person who can detect early warning signs, assess whether the circumstances described in the SBD obtain, and initiate involuntary hospital admission and treatment. A person of trust can be a partner (formal or informal), a family member, or a friend. Including a person of trust in the process of drafting an SBD can create a shared understanding of service users’ medical condition, their preferences, and helpful interventions in a crisis.

*Avoidance of involuntary hospital admission:* Although service users can use SBDs to give advance consent to involuntary hospital admission, SBDs can prevent involuntary admissions in two ways. First, service users can use their SBD to request intensified community support services when they manifest early warning signs. Second, when service users refuse hospital admission, persons of trust and professionals can use the SBD to remind service users of their considered preferences and persuade them to accept hospital admission voluntarily.

*Addressing trauma:* During the process of drafting an SBD, service users reflect on experiences of involuntary admission and treatment in the past and incorporate these experiences in a narrative about how they prefer to be treated in the future. Although this process can be an emotionally stressful process, going through it can be helpful in addressing past trauma and achieving a sense of acceptance and empowerment.

*Destigmatization of involuntary treatment:* Involuntary treatment is subject to social stigma, and part of what is stigmatizing about involuntary care is arguably that the agency of service users is denied. SBDs allow service users to stay in charge of future involuntary treatment, and the drafting process provides an occasion to discuss these matters openly with others.

*Increase of professionals’ confidence:* Professionals often experience moral distress in relation to involuntary treatment because they are unsure about whether they act in ethically justifiable ways. SBDs can reduce moral distress in professionals by offering concrete guidance on involuntary treatment and providing professionals with assurance that SBD-compliant treatment is in accord with service users’ considered preferences.

*Relief for proxy decision-makers:* When a service user lacks mental capacity, a proxy decision-maker should aim to make treatment choices based on the fundamental values and convictions of the service user or in her (subjective) best interests. Proxy decision-makers often experience this as a burdensome task because they are unsure about how the service user would want to be treated in the circumstances. SBDs can provide clarity and relief to proxy decision-makers by offering concrete guidance on this question.

### Challenges of SBDs

Stakeholders perceived various challenges of SBDs. Challenges on which the studies converged are summarized in Table 4.

**Table 4.** Perceived challenges of SBDs

Challenges of SBDs
Lack of awareness and knowledge
Lack of support
Undue influence
Inaccessibility during crisis
Lack of cross-agency coordination
Problems of interpretation
Difficulties in capacity assessment
Restricted therapeutic flexibility
Scarce resources
Disappointment due to noncompliance
Outdated content

Abbreviation: SBDs, self-binding directives.

*Lack of awareness and knowledge:* Stakeholders consistently reported a lack of awareness and knowledge of SBDs among service users and professionals alike. This concerns not only a lack of professional education and training but also a lack of normative guidance, resource and information materials, and SBD templates for service users and professionals.

*Lack of support:* Drafting an SBD is a complex process in which service users need informational, emotional, and administrative support. They require support in understanding the potential benefits and risks of the available treatment options, processing past experiences and handling the emotions generated, and registering and distributing the SBD. Support is mostly lacking in current clinical practice.

*Undue influence:* Service users, relatives, and professionals can have conflicting interests, and relatives and professionals might exert undue influence on the service user in the process of drafting an SBD.

*Inaccessibility during crisis:* For an SBD to be applied successfully, its content should be accessible to a close one who can detect early warning signs and the treatment team of the responsible hospital. Under real-life conditions, the SBD might not be sufficiently accessible to the responsible persons.

*Lack of cross-agency coordination:* Many agencies can be involved in initiating and carrying out involuntary admissions (e.g., the police and social services, community and primary care, acute and inpatient care services). These agencies may not be familiar with or not have access to SBDs or lack competency in handling them, while communication between agencies may be difficult.

*Problems of interpretation:* SBD instructions might not be sufficiently clear and might give rise to problems of interpretation. Parties involved may disagree about the circumstances in which the SBD is meant to apply or about the meaning of the treatment preferences described in the SBD.

*Difficulties in capacity assessment:* It can be difficult to determine whether the service user lacks mental capacity when the service user exhibits the early warning signs described in the SBD. Since overriding treatment refusals of service users who have mental capacity would be impermissible, it can accordingly be difficult to determine whether an SBD applies and whether the instructions included in it must be followed.

*Restricted therapeutic flexibility:* If SBDs contain detailed treatment instructions, the flexibility of professionals in providing effective treatment may be limited. This can be problematic when situations arise which were not anticipated in the drafting process. Concerns about limited therapeutic flexibility were raised predominantly by professionals working in Germany and less by those working in the Netherlands and the UK.

*Scarce resources:* Professionals may not have sufficient time to facilitate the drafting process, and the required time can likely not be reimbursed. Scarce resources can also be a factor in giving effect to SBDs. There may be no beds available in the designated hospital, or the professional who was involved in the drafting process may be unavailable. Involuntary admission based on an SBD when there is a scarcity of beds or personnel may also come at the expense of others who are in stronger need. Concerns about scarce personnel were raised in all three countries, whereas concerns about limited availability of hospital beds were more prominent in the Netherlands and the UK than in Germany.

*Disappointment due to noncompliance:* Failure to comply with SBDs on the part of the treatment team may result in disappointment among service users, and this is likely to have a negative impact on the therapeutic relationship.

*Outdated content:* The content of SBDs may be outdated and fail to reflect service users’ considered preferences if the SBD is not updated regularly.

## Discussion

Notwithstanding predominantly inductive research designs and legal and clinical background differences between the three countries, findings from the studies on stakeholders’ perspectives on the opportunities and risks of SBDs converged on many points.

Substantial differences were found primarily in relation to concerns about limited therapeutic flexibility and limited availability of hospital beds. In Germany, concerns about limited therapeutic flexibility were more prominent and concerns about limited availability of hospital beds less prominent than in the Netherlands and the UK. A possible explanation of the former finding is that in Germany, unlike in the latter two countries, it is not legally permitted for professionals to override PADs by reference to the welfare or best interests of the service user. A possible explanation of the latter finding is that the Netherlands and the UK have lower bed-to-inhabitants ratios than Germany: in 2021, the UK had 0.34 and the Netherlands had 0.79 psychiatric beds per 1000 inhabitants, as compared to 1.30 in Germany [40].

A notable finding of our international comparison is that stakeholders did not confirm the fundamental ethical and legal concerns raised by ethicists and legal scholars. Stakeholders voiced few or no worries about an increase of coercion or the invalidity of SBDs due to a lack of identity between past and present self or outdated consent – all of which feature prominently in the ethical and legal literature [26–28]. The possibility of undue influence during SBD completion, however, was an important challenge from the conceptual literature which stakeholders raised.

Stakeholders rather focused on challenges of a practical nature. It must be taken into account, however, that some of these practical challenges (e.g., limited therapeutic flexibility and scarce resources) can have ethical implications. Most stakeholders had a positive overall evaluation of SBDs, either because they thought that the benefits of SBDs outweigh their risks or because they thought that the associated challenges can be addressed through the implementation of safeguards. The possibility of undue influence during SBD completion, for example, can be addressed by including a person of trust (e.g., a relative) or a neutral party (e.g., a peer support worker) in the drafting process. Recommendations for safeguards and due care criteria to address challenges in SBD implementation have been given in the literature [41].

### Strengths and limitations

This is the first international comparison on SBDs to date, and it is based on comprehensive qualitative and quantitative stakeholder research. The generalization of results might be limited by the fact that conclusions are based on findings from three Western European countries. The comparison showed, however, that the findings of the studies converged on many points despite significant differences in mental health legislation, mental health systems, and professional cultures between the countries. This suggests that our findings may be appropriate in other high-income countries with well-developed mental health laws and services. Application in countries that lack one or more of these characteristics should be context-sensitive and consider viability and feasibility under the relevant mental health laws and services.
